# Anxiety and Obesity in Children: Anti-oxidative Medications and Hyperbaric Oxigenation Therapy

**DOI:** 10.1192/j.eurpsy.2023.726

**Published:** 2023-07-19

**Authors:** D. Labunskiy, G. Kukina, M. Kolmykova, S. Kiryukhina

**Affiliations:** 1 Ogarev Mordovia State University, Saransk, Russian Federation; 2 Ogarev Mordovia State University, Ogarev Mordovia State University, Saransk, Russian Federation

## Abstract

**Introduction:**

Obesity is a grave problem in pediatrics and child psychiatry. It was revealed that in many cases obesity has comorbidity with different mental disorders. Besides a number of endocrine disturbances, immune system deficiency and oxidative stress with free radical oxidation take part in pathogenesis of these phenomena.

**Objectives:**

The essence of the therapeutic effect of hyperbaric oxygen lies in the fact that under the influence of elevated barometric pressure, oxygen saturation of body fluids increases: blood, interstitial fluid, which deliver oxygen to organs and tissues, increasing oxygen supply to cells by 5-10 times and eliminating hypoxia (oxygen starvation tissues). Hyperbaric oxygen, normalizing blood circulation in tissues and metabolic processes at the cellular level, has a number of effects that favorably affect the patient’s condition: promotes the formation of a new vascular network in areas where it is damaged or insufficient, has anti-edematous, anti-inflammatory and wound healing effects, has an immunocorrective effect , normalizes hormonal status, enhances the action of a number of pharmacological agents. The aim of our research was to study effects of HBO on children suffering from anxiety and obesityas a result of the mental instability? S;eep disturbances and immune instability

**Methods:**

Sessions of hyperbaric oxygenation were carried out at a pressure of 1.6 ATA: the first session lasted 45 minutes, subsequent sessions was 30 minutes. After HBO, each of the patients during the day showed a steady increase in the saturation index, which persisted until the morning of the next day. An analysis of fluctuations in the saturation index during the day showed its decrease in all patients to a minimum value at 8 am and its general dynamic increase from the beginning of hyperbaric oxygenation. Parameters of immune status, including serum concentrations of IgA, IgM and IgG, circulating immune complexes were studied in pediatric patients, aged from 10 to 14 years old, 42 girls and 34 girls. Mapping EEG was used for evaluation of electric brain activity.

**Results:**

It was found that HBO treatment normalized mental state: patients demonstrated less anxiety and overeating habits, тormalization of sleep, positive dynamics of sleep, reflected in the EEG pattern in the form of alpha waves. Immune reactions were changed: IgA, IgM and IgG, were increased and CIC serum level decreased.

**Conclusions:**

The use of antioxidant therapy in combination with psychopharmacological drugs has long been a well-established method of treatment for a variety of psychopathological conditions. The inclusion of hyperbaric oxygenation in this complex also helps to achieve more distinct results in terms of weight loss in obesity and normalization of the mental state, improved sleep and significant immunocorrection.

**Disclosure of Interest:**

None Declared

